# Solid-phase microextraction and on-fiber derivatization for assessment of mammalian and vegetable milks with emphasis on the content of major phytoestrogens

**DOI:** 10.1038/s41598-019-42883-7

**Published:** 2019-04-25

**Authors:** Antonella Aresta, Pietro Cotugno, Carlo Zambonin

**Affiliations:** 1 Department of Chemistry, University of Bari “Aldo Moro”, Via Orabona 4, 70125 Bari, Italy; 2 CONISMA, Piazzale Flaminio 9, 00196 Roma, Italy

**Keywords:** Nutrition, Public health

## Abstract

A new solvent-free method for the simultaneous determination of some major phytoestrogens (equol, enterodiol, daidzein, genistein, glycitein) in different commercial milks (cow, goat and soy-rice) was developed. After solid phase microextraction, performed by direct immersion of a 65 μm-polydimethylsiloxane–divinylbenzene fiber in diluted (1:100 with 0.2% formic acid - 30% sodium chloride) milk samples (18 °C for 20 min under stirring), a direct on-fiber silylation with N,O-bis (trimethylsilyl)trifluoroacetamide) containing 1% trimethylchlorosilane (70 °C for 20 min) was performed prior to gas chromatography–mass spectrometry analysis. Since the target compounds were determined as aglycones, the hydrolytic removal of the aglycone from the glycosides was performed. The method permitted the determination of the target analytes in all the considered milk samples as well as the detection of some major amphipathic fats indicating that the approach could potentially be applied in the future for further applications, such as milk profiling.

## Introduction

Milk is the primary source of nutrition for young mammals. It contains important nutrients which are essential for the growth of infant, such as lactose, fats, proteins, vitamins, and salts^1^. Due to its taste and beneficial properties, humans often continue to consume milk from other species. However, some individuals develop lactose intolerance or allergies to some of its components. Consequently, some people choose to consume non-animal milks, such soy, rice, almond, or coconut, that do not contain lactose, dairy proteins or cholesterol^2^. Furthermore, vegetable milks are greatly consumed in various cultures, both as a regular drink or milk replacement.

Vegetable milks contain high levels of phytoestrogens, a class of poly-phenolic compounds characterized by high structural similarity to 17β-estradiol^3^. Phytoestrogens have significant effects for the prevention of menopausal symptoms and several diseases, including breast, endometrial, and prostate cancers^4,5^. In plants, they are usually found in conjugated forms as glycosides. Two main classes of phytoestrogens are isoflavones and lignans.

Daidzin, genistin and glycitin are major isoflavones found in a wide variety of plant, in particular soybean and clover^5–7^. They are metabolized in the intestine to their aglycone forms, daidzein, glycitein and genistein, respectively, that possess higher estrogenic power^3,8^. It has been recently stated that also cow’s milk may contain appreciable amounts of isoflavones arising from the ingestion of forage by cows^9^.

Lignans are a large class of secondary metabolites in plants that have numerous biological effects in mammals, including antitumor and antioxidant activities. Equol is a lignan metabolized from daidzein that possess longer half-life, higher antioxidant properties and affinity for the estrogen receptors than its precursor^10^. In addition, it has protective effects against UV-induced DNA damages^11^. Equol concentration ranges from 10 to 411 ng/mL in cow’s milk, while it is not a normal constituent of plant milks^12,13^. Enterodiol is a mammalian lignan with antitumor activity mainly produced in the colon by the action of gut bacteria on its glycosides matairesinol^14^.

Phytoestrogens analysis in different matrices, such as food and biological fluids, have been mainly carried out by gas chromatography after derivatization, liquid chromatography (LC) (both with and without mass detectors), capillary electrophoresis (CE) and immunoassays^9,15–17^. Most of the methods, that have been recently reviewed^9,17^, require large sample volume and time consuming multi-step sample preparation, thus faster and simpler approaches are highly desirable.

Recently, solid-phase microextraction (SPME) coupled with LC-UV/DAD, has been successfully used for the extraction and analysis of some major isoflavones from bovine milk and soy derived products^18,19^. The advantages of the SPME approach are well established, i.e. simplicity, low use of organic solvents, low cost and easy automation. Thus, it could be used, after a derivatization step, for the determination of phytoestrogens in milk samples also in combination with gas chromatography – mass spectrometry (GC-MS). SPME-GC-MS was used to quantify free short chain fatty acids (CFA-C12) in milk samples^20^, while the possibility to perform a post-fiber derivatization prior to GC analysis of polar analytes was reported by Pan and Pawliszyn, which showed the improvement obtained on GC separation, detection and quantification of fatty acids in water^21^, and by Ferrer *et al*., that performed a fragmentation study of phytoestrogens as their trimethylsilyl derivatives and their identification in soy milk and wastewater^22^.

In this paper, SPME and direct on-line derivatization followed by GC-MS analysis have been used for first time for the simultaneous determination of major phytoestrogens (equol, enterodiol, daidzein, genistein, glycitein) and was validated in terms of linearity, limits of detection, quantification and precision. Then, the procedure was successfully used for the determination of the analytes in different commercial milk samples (cow, goat and soy-rice), before and after β-glucosidase deconjugation. The method permitted also the detection free fatty acids (FFA), their monoglycerides (MAG) and cholesterol, indicating that it could potentially be used for different applications, such as milk profiling.

## Results and Discussion

SPME of the target analytes was performed using recently optimized^18^ conditions, i.e. 20 min at room temperature directly exposing a 65 μm PDMS-DVB fiber to a 0.2% formic acid - 30% NaCl solution under constant magnetic stirring. As far as the derivatization of the analytes is concerned, three different procedures can be used in combination with SPME: derivatization in the GC injection port, *in-situ* derivatization and on-fiber derivatization^23–25^. In the first case, derivatization reactions occur on the SPME fiber at high temperature in the GC injection port. This approach is characterized by simplicity, efficiency and low consumption of potential poisonous reagents^25^. The *in-situ* derivatization involves the addition of the derivatizing agent to the sample matrix and the subsequent SPME of the derivatized analytes from the medium (solution or headspace), even if the presence of the agent in the sample can negatively affect the extraction. In the present work, the direct on-fiber silylation with BSTFA was used since, as already reported, it was found to be effective for the analysis of estrogens and anabolic steroids^26^. After the extraction, the SPME fiber was dried under nitrogen to avoid the hydrolysis of the derivatizing agent, transferred into a heated and sealed vial and exposed to the vapors of the derivatizing agent for a given time.

The effects of time, temperature and derivatizing agent volume on the derivatization reaction efficiency were carefully investigated performing the relevant experiments individually for each analyte. It is worth noting that all the analytes have various hydroxyl groups, thus they could in theory generate multiple derivatives, resulting in reduced performances of the method. In fact, short derivatization times produced different reaction products for all the analytes. Of course, the formation of a single derivative should be preferred to obtain the highest sensitivity. Eventually, it was found that the optimal conditions were achieved by using 5 μL of BSTFA - 1% TMCS at 70 °C for 20 minutes. In these conditions, that were then used throughout the work, the best reaction yields and the formation of one derivative for each analyte were observed. The fiber was finally placed into the GC injector at 250 °C for a period of 10 min for analytes desorption. The estimated average percentage carryover was 6.6 ± 5.8%. The whole procedure was well tolerated by the fiber, that was usable for at least 60 steps of extraction/derivatization.

**Figure 1:**
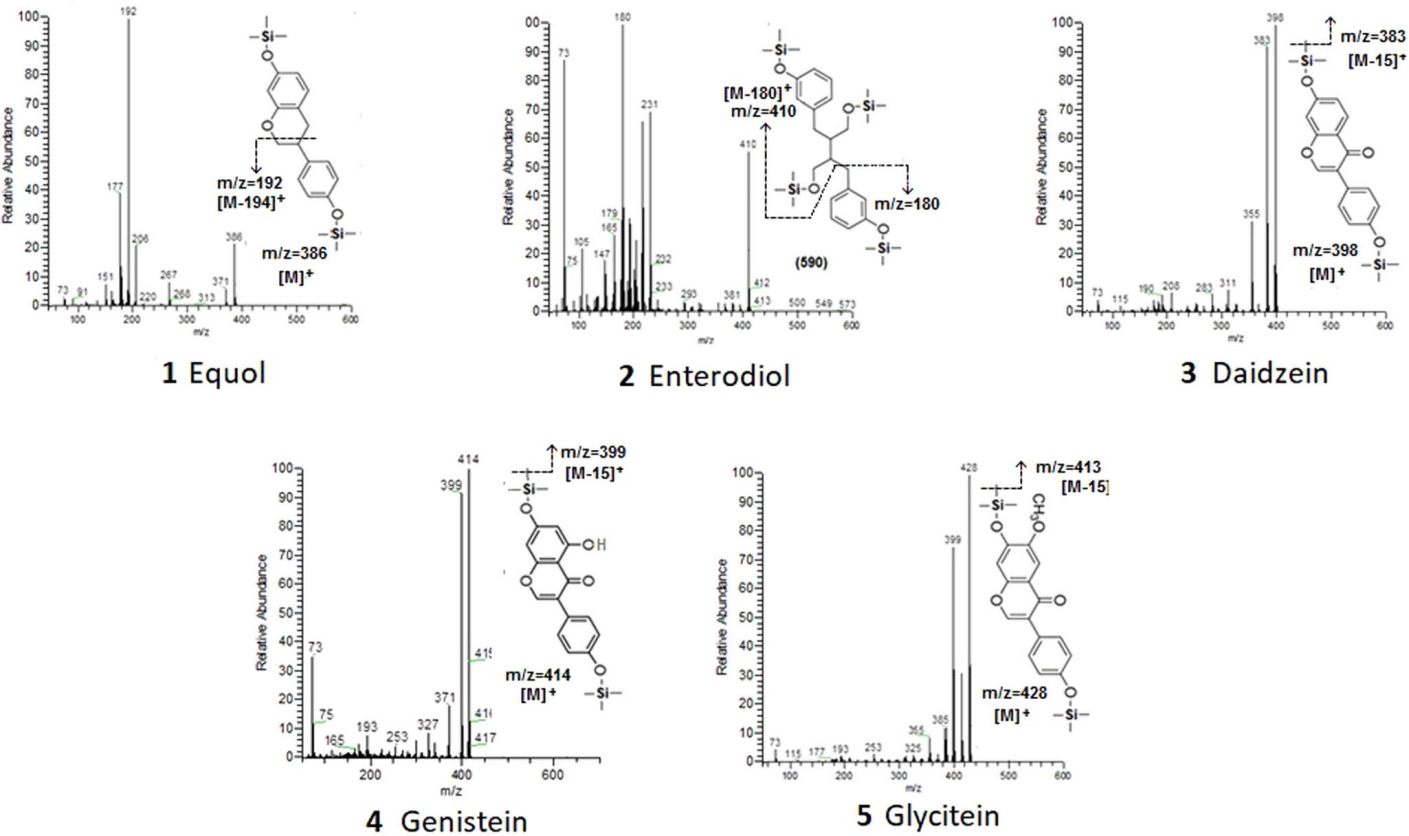
Mass spectra of the TMS derivates of the target compounds.

Figure 1 reports the mass spectra of the TMS derivates obtained for the target compounds, while Table 1 resumes their retention time, molecular weight (before and after derivatization), main m/z ions observed in the mass spectra and those used for quantification.

**Table 1:** Retention time, molecular weight (before and after derivatization), main m/z ions and m/z ions used for quantification, for each analyte.

Compound in elution order	Retention time (R.T., min)	MW	TMS groups	MW of the TMS derivate	Main observable m/z ions	Quantification m/z ions
Equol	33.70 ± 0.08	242	2	386	177, 192, 371, 386	192
Enterodiol	34.65 ± 0.09	302	4	590	105, 180, 410, 500	180
Daidzein	39.75 ± 0.09	254	2	398	184, 383, 398	398
Genistein	40.48 ± 0.08	270	2	414	371, 399, 414	414
Glycitein	42.80 ± 0.09	284	2	428	398, 413, 428	428

The base peak in the mass spectrum of the di-TMS derivate of equol was the m/z ion 192, produced by the cleavage of the chromanol ring, while the [M]^+^ and [M-15]^+^ ions were characterized by low intensities. The fragmentation of the tetra-TMS derivate of enterodiol produced characteristic ions such as the m/z 180 ([M-410]^+^), arising from the cleavage of the aromatic ring of the TMS derivate^27^, and the m/z 410 [M-180]^+^. The mass spectra of the di-TMS derivates of daidzein, genistein and glycitein were characterized by molecular ions [M]^+^ as base peaks (m/z 398, 414 and 428, respectively) and intense [M-15]^+^ fragment ions corresponding to the loss of a methyl group from the molecular ion (m/z 383, 399 and 413, respectively)^22^. Therefore, the m/z ions 192, 180, 398, 414 and 428 were used as quantification ions.

**Figure 2:**
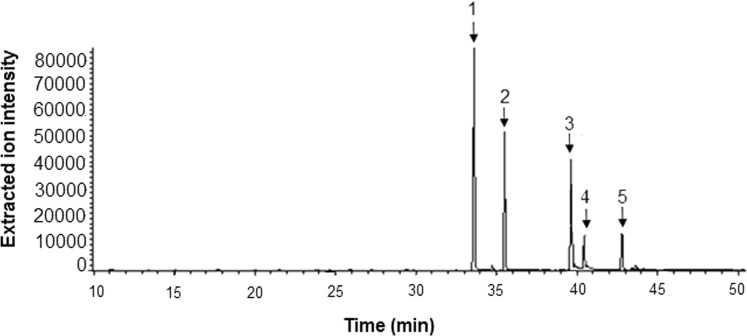
GC-MS extracted-ion chromatogram of a standard solution (1 μg mL^−1^) of the TMS derivates of the target compounds: 1) equol; 2) enterodiol; 3) daidzein; 4) genistein; 5) glycitein.

Figure 2 reports a GC-MS extracted-ions chromatogram (XIC) relevant to a standard solution (1 μg mL^−1^) of the TMS derivates of the target analytes. The XIC acquisition mode was used in the work since no significance increases in sensitivity were found acquiring the samples in selected ion monitoring mode (SIM).

The method was validated in terms of linearity, limits of detection (LOD) and quantification (LOQ), and precision. The results showed good linearity for all the analytes within the range of application with correlation coefficients always better than 0.999. LOD and LOQ ranged from 0.02 ng mL^−1^ (equol) to 0.28 ng mL^−1^ (genistein) and from 0.08 ng mL^−1^ (equol) to 0.95 ng mL^−1^ (genistein), respectively, which are comparable to those already reported in the literature. The linear ranges, LOD and LOQ are reported in Table 2. The precision of the method (expressed as RSD %) was always lower than 9.8%, which complies with the acceptance criteria. No significance differences were observed between the within-day and day-to-day reproducibility according to an F-test. The relevant results are reported in Table 3.

**Table 2:** Linear ranges, LOD and LOQ obtained for the analytes.

Compound	Linear range (ng mL^−1^)	LOD (ng mL^−1^)	LOQ (ng mL^−1^)
Daidzein di-TMS	0.10–1000	0.03	0.10
Genistein di-TMS	0.90–2000	0.28	0.95
Glycitein di-TMS	0.20–1000	0.08	0.25
Equol di-TMS	0.08–1000	0.02	0.08
Enterodiol tetra-TMS	0.50–2000	0.15	0.50

**Table 3:** Within-day (n = 5) and between-days (n = 5, for five days) precision.

Compound	within-day			between-days		
	0.01 (µg mL^−1^)	0.1 (µg mL^−1^)	1 (µg mL^−1^)	0.01 (µg mL^−1^)	0.1 (µg mL^−1^)	1 (µg mL^−1^)
daidzein	9.4%	9.2%	9.5%	9.5%	9.3%	9.7%
genistein	9.6%	9.5%	9.7%	9.8%	9.6%	9.8%
glycitein	9.6%	9.5%	9.6%	9.7%	9.6%	9.8%
equol	9.8%	9.6%	9.7%	9.8%	9.8%	9.8%
enterodiol	9.7%	9.6%	9.8%	9.8%	9.7%	9.8%

**Figure 3:**
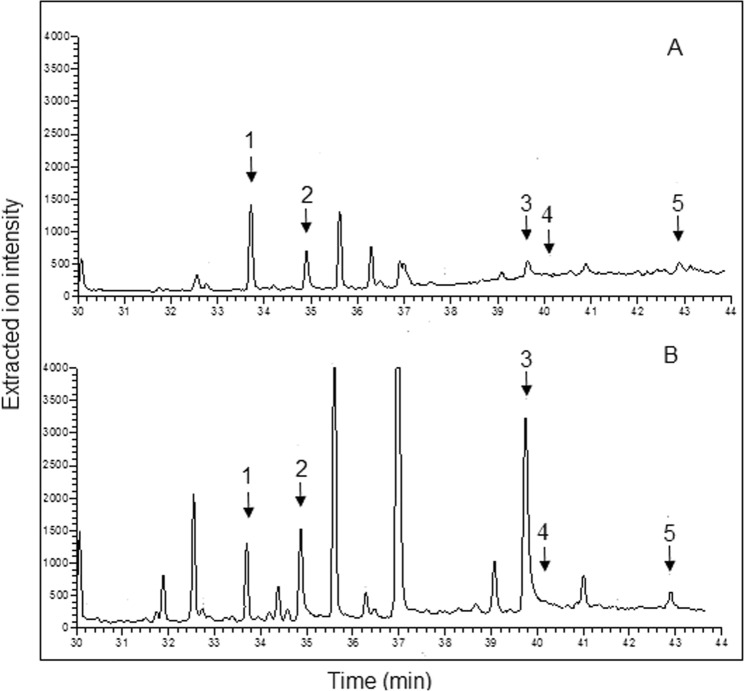
GC-MS XICs (time window 29–49 min) relevant to the analysis of a not digested (A) and digested (B) cow milk sample. 1) equol; 2) enterodiol; 3) daidzein; 4) genistein; 5) glycitein.

The developed method was then applied to the determination of the analytes in different commercial milks (cow, goat and soy-rice). Since the target compounds were determined as aglycones, β-glucosidase was added to each sample for the hydrolytic removal of the aglycone from the glycosides. It was recently reported^28^ that the concentration of phytoestrogen could be likely overestimated if hydrolysis is performed by means of the β-glucuronidase originating from *Helix pomatia*. In fact, this enzyme is contaminated by several phytoestrogen isoflavones such as genistein and daidzein and their metabolite equol. Consequently, in the present work β-glucosidase from almonds was selected, analyzed and found to be not contaminated at all with the target analytes (Fig 1S). Thus, the use of this enzyme permitted to avoid the risk of contamination and consequent overestimation of the analytes. All the chromatograms were acquired before and after the enzymatic digestion to assess both the free and total amount of each analyte. Figure 3 reports, for instance, the GC-MS XICs (time window 29–49 min) relevant to the analysis of, respectively, a not digested (A) and digested (B) cow milk sample. As apparent, the chromatograms were characterized by the presence of a significance number of peaks arising from interfering compounds that were well resolved from the target phytoestrogens.

Quantitation was performed by the standard addition method. This decision was taken considering the high complexity of the sample matrices, the low levels of the analytes in the milks, and the fact that there was no ideal blank sample available. The average concentrations of the analytes found in milk samples before and after β-glucosidase deconjugation are listed in Table 4.

**Table 4:** Average concentrations of the analytes found in selected milk samples before and after β-glucosidase deconjugation.

Compound	[Cow milk] (μg mL^−1^)		[Goat milk] (μg mL^−1^)		[Soy-rice drink] (μg mL^−1^)	
	Before	After	Before	After	Before	After
daidzein	0.010 ± 0.01	0.087 ± 0.01	0.025 ± 0.01	0.055 ± 0.01	1.562 ± 0.09	37.053 ± 1.89
genistein	nd	nd	nd	nd	0.192 ± 0.02	27.140 ± 1.66
glycitein	0.120 ± 0.01	0.270 ± 0.02	nd	nd	0.096 ± 0.01	1.203 ± 0.10
equol	0.159 ± 0.01	0.210 ± 0.01	0.212 ± 0.02	0.335 ± 0.03	nd	nd
enterodiol	0.216 ± 0.02	0.310 ± 0.02	<LOD	<LOD	nd	nd

The accuracy of the procedure was demonstrated by the value of the overall average recovery, which was 85.5% with RSD% always less than 12.6% for all samples at all the tested concentrations levels. Table 5 reports, as an example, the recoveries obtained in the case of bovine milk. Similar results were obtained in the case of the other considered samples.

**Table 5:** Recovery data obtained in the case of bovine milk samples.

Compound	Before enzymatic deconjugation (n = 3)					
	Added (µg mL^−1^)	Rec (%)	Added (µg mL^−1^)	Rec (%)	Added (µg mL^−1^)	Rec (%)
daidzein	0.01	83 ± 13	0.05	85 ± 9	0.1	85 ± 8
genistein	0.1	84 ± 12	0.5	85 ± 10	1.0	87 ± 11
glycitein	0.12	89 ± 9	0.6	90 ± 5	1.2	89 ± 7
equol	0.16	88 ± 8	0.8	86 ± 6	1.6	85 ± 10
enterodiol	0.22	82 ± 11	1.1	80 ± 6	2.2	85 ± 5
**Compound**	**After enzymatic deconjugation (n** = **3)**					
	**Added (µg mL** ^**−1**^ **)**	**Rec (%)**	**Added (µg mL** ^**−1**^ **)**	**Rec (%)**	**Added (µg mL** ^**−1**^ **)**	**Rec (%)**
daidzein	0.1	84 ± 10	0.5	87 ± 9	0.9	89 ± 8
genistein	0.1	80 ± 9	0.5	82 ± 11	1.0	85 ± 12
glycitein	0.3	86 ± 8	1.5	88 ± 9	3.0	85 ± 11
equol	0.2	89 ± 9	1.0	90 ± 10	2.0	85 ± 10
enterodiol	0.3	83 ± 13	1.5	85 ± 9	3.0	85 ± 10

The presence of daidzein in all selected samples was expected, as well as that of equol and enterodiol in ruminant milks. Their estimates agreed with previously reported data^9,18^. The high sensitivity of the method permitted the determination of glycitein for the first time in cow and soy-rice milk. As expected, genistein was determined at high concentration only in soy-rice milk.

It is worth noting that the developed method also permitted the extraction/derivatization and detection of some major amphipathic fats, such as free fatty acids (FFA), their monoglycerides (MAG) and cholesterol, indicating that the method could potentially be applied in the future for different applications, such as milk profiling. The fats were identified through the National Institute of Standards and Technology (NIST) library of the GC-MS instrument and are listed in Table 6.

**Table 6:** Compounds tentatively identified through the NIST library.

Retention time (min)	Derivative Formula	Category, Lipid Numbers	Prob.
12.44 ± 0.08	C_15_H_32_O_2_Si	FFA, C12:0	>81.6
17.42 ± 0.08	C_17_H_36_O_2_Si	FFA, C14:0	>71.9
21.57 ± 0.09	C_19_H_38_O_2_Si	FFA, C16:1	>88.9
22.20 ± 0.08	C_19_H_40_O_2_Si	FFA, C16:0	>93.9
25.89 ± 0.08	C_21_H_40_O_2_Si	FFA, C18:2	>89.6
26.02 ± 0.08	C_21_H_42_O_2_Si	FFA, C18:1	>90.1
26.6 ± 0.09	C_21_H_44_O_2_Si	FFA, C18:0	>79.8
30.58 ± 0.09	C_23_H_48_O_2_Si	FFA, C20:0	>80.4
30.68 ± 0.08	C_23_H_50_O_4_Si_2_	MAG, C14:0	>91.6
33.38 ± 0.09	C_25_H_54_O_4_Si_2_	MAG, C16:0	>96.3
36.55 ± 0.08	C_27_H_58_O_4_Si_2_	MAG; C18:0	>89.0
40.4 ± 0.09	C_29_H_62_O_4_Si_2_	MAG; C20:0	>78.9
46.85 ± 0.08	C_30_H_54_OSi	Sterol, cholesterol	>89.0

## Conclusions

SPME, with direct on-fiber derivatization, coupled to GC-MS was successfully used for the first time for the simultaneous determination of target phytoestrogens in cow, goat and soy-rice milk samples before and after β-glucosidase deconjugation, as demonstrated by the analysis of several real samples.

The method was also able to detect in the same samples some major free fatty acids, their monoglycerides and cholesterol, easily identified by NIST library. The data were useful for highlighting differences between beverages by comparison of their lipidic profiles and suggested that the approach could potentially be applied for different applications, such as milk profiling.

## Methods

### Chemicals

All chemicals were from Sigma Aldrich (Milano, Italy). (±)-Equol (4′,7-dihydroxyisoflavane), enterodiol (3,3′-[2,3-Bis (hydroxymethyl) butane-1,4-diyl] diphenol), daidzein (4′,7-dihydroxyisoflavone), genistein (4′,5,7-trihydroxyisoflavone), glycitein (4′,7-dihydroxy-6-methoxyisoflavone) stock solutions (1 mg mL^−1^) were prepared in a methanol/dimethyl sulfoxide mixture (5:5, v/v) and stored at −20 °C in the dark. Working solutions were prepared daily in 0.2% formic acid with 30% sodium chloride. BSTFA (N,O-bis (trimethylsilyl) trifluoroacetamide) containing 1% TMCS (trimethylchlorosilane) was stored at room temperatures in the dark. Before use, the enzymatic solution for glycosides hydrolysis was obtained by dissolving 10 mg of β-glucosidase from almonds (≥2 units/mg solid, Sigma) in acetate buffer (0.1 M, pH 5.0).

### SPME: Extraction and derivatization

Extraction was performed by immersing the SPME fiber (65 μm-polydimethylsiloxane–divinylbenzene, PDMS-DVB) directly into a 1.5 mL vial, sealed with hole-cap and PTFE/silicon septum and prefilled with the working standard solution, for 20 min at ambient temperature (18 °C) under constant magnetic stirring. Then, the fiber was removed and dried using a nitrogen stream for 2 minutes. Finally, it was inserted into the headspace of a vial (250 μL) containing 5 μL of BSTFA – 1% TMCS and placed into a metallic block heated at 70 °C for 20 minutes. After derivatization, the analytes were desorbed in the injection port of the GC in splitless mode at 250 °C for 3 minutes.

### Gas chromatography–mass spectrometry

GC-MS analysis was performed using a Finnigan TRACE GC Ultra Gas chromatograph equipped with a Finnigan PolarisQ ion trap mass spectrometer (Thermo Finnigan). A TR-5MS fused silica capillary column (30 m × 0.25 μm i.d., 0.25 μm film thickness, Thermo scientific) was employed. The column temperature program was set as follows: 130 °C (3 min), 10 °C min^−1^ to 180 °C, then increased 4 °C min^−1^ to 280 °C and held for 10 min at 280 °C. The carrier gas was helium (99.999%) at a constant flow of 1 mL min^−1^. The inlet port, operating in splitless mode (3 min), was set at 250 °C, while the temperature of the transfer line was 280 °C. The mass spectrometer was operated in the electron impact positive (EI^+^) ionization mode with the source set at 220 °C. The electron energy was 70 eV and the filament current 150 μA. Mass spectra were acquired in the m/z range 50–600 (0.55 scans s^−1^). Analytes were detected using extracted-ion chromatograms, extracting the m/z ions 180, 192, 398, 414 and 428.

### Linear Range, Detection Limits and Precision

Calibration curves were calculated for all the analytes. The limits of detection (LODs) and quantitation (LOQs) were calculated as 3- and 10-fold the standard deviation of the intercept of the calibration curves. The within-day (n = 5) and between-days (n = 5, for five days) precision of the method were evaluated at three different concentrations (0.01, 0.1 and 1 µg mL^−1^).

### Collection and preparation of the milk samples

Two soy-rice (a mixture of soymilk with amazake, a traditional Japanese porridge made from fermented brown rice) and four semi-skimmed (two cows and two goats) milks were purchased from local stores and stored in aliquots (250 μL) at −20 °C. Each aliquot was thawed at room temperature before analysis, diluted 1:100 with 0.2% formic acid - 30% NaCl, subjected to the developed SPME extraction/derivatization procedure and analyzed by GC-MS using the optimized conditions. For the hydrolysis of the conjugates, 250 μL of the enzymatic solution (380 units) was added to 250 μL of milk and incubated overnight at 37 °C. Finally, 30 μL were diluted 1:50 with 0.2% formic acid - 30% NaCl before being processed and analyzed. In order to check the purity of β-glucosidase from almonds, 250 μL of the enzymatic solution (380 units) was added to 250 μL of water (Fig 1S). Then, 30 μL were diluted 1:50 with 0.2% formic acid - 30% NaCl, subjected to the developed SPME extraction/derivatization procedure and analyzed by GC-MS using the optimized conditions.

Quantifications were performed by the standard addition method. Briefly, 0.475 mL of original milk was taken and transferred to four tubes of which one was kept as blank sample and further added with 0.025 mL of a methanol/dimethyl sulfoxide mixture (5:5, v/v), while the others received 0.025 mL of an accurate standard mixture, this to minimize the interferences of the solvent with the results. We used three suitable standard mixture, each containing the isoflavones at levels of 20, 100, and 200-fold those estimated in the blank sample through the external calibration curve, to spike the milk samples at 1, 5 and 10 times the original levels, respectively. All samples were equilibrated at 37 °C in a water bath for 30 minutes before being diluted 1:100 for SPME-HPLC analysis. Three replicas for each concentration, including blank, were made. The exact concentrations of isoflavones in each sample were estimated on the basis of the absolute values of the x-intercepts of the (unweighted) regression lines obtained.

The accuracy was checked by conducting a recovery study. Recovery (%) of each compound was obtained by comparing the peak areas of the analytes in spiked samples at 1, 5, and 10-fold original levels with the peak areas of equivalent mixed standards solutions, carrying out each measurement in triplicate. The addition was performed on the original milk samples, as described above for the standard addition method.

### Ethical Approval

This article does not contain any studies with human or animal subjects.

## Supplementary information

Fig 1S
