# Supplementary material for: Solid-phase microextraction and on-fiber derivatization for assessment of mammalian and vegetable milks with emphasis on the content of major phytoestrogens
Source: Sci Rep. 2019 Apr 25;9:6398. doi: 10.1038/s41598-019-42883-7 (PMC6484104; doi:10.1038/s41598-019-42883-7)
Supplement: Supplementary file 1 — Fig 1S [file 41598_2019_42883_MOESM1_ESM.pdf]

Solid-phase microextraction and on-fiber derivatization for assessment of mammalian and vegetable milks with emphasis on the content of major phytoestrogens

Antonella Aresta, Pietro Cotugno and Carlo Zambonin.

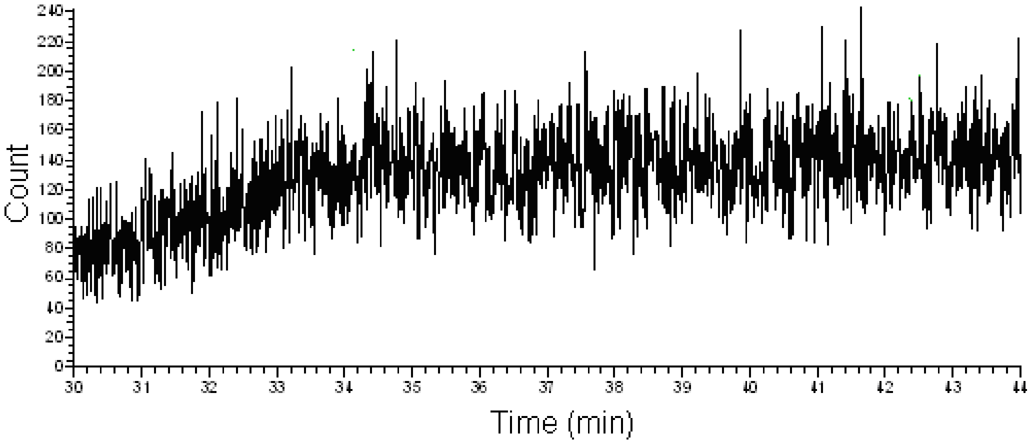

Fig 1S. GC-MS XICs (time window 29-49 min) relevant to the analysis of β-glucuronidase from almonds.
